# Bridging pleiotropic mechanisms in leprosy type-1 reactions and neurodegenerative diseases

**DOI:** 10.1038/s41598-025-30734-7

**Published:** 2025-12-22

**Authors:** Vinicius M. Fava, Jônatas Perico, Marianna Orlova, Monica Dallmann-Sauer, Yong Zhong Xu, Nguyen Van Thuc, Vu Hong Thai, Andrea F. Belone, Ana Carla P. Latini, Erwin Schurr

**Affiliations:** 1 Program in Infectious Diseases and Global Health, The Research Institute of the McGill University Health Centre, Montreal, Canada; 2 Faculty of Medicine, Department of Medicine, McGill International TB Centre, McGill University, Montreal, Canada; 3 Present Address. Canadian Centre for Computational Genomics (C3G) McGill University, Montreal, Canada; 4 Instituto Lauro de Souza Lima, Bauru, São Paulo, Brazil; 5 Program in Tropical Diseases, São Paulo State University (Unesp), Medical School, Botucatu, São Paulo, Brazil; 6 Aligning Science Across Parkinson’s (ASAP) Collaborative Research Network, Chevy Chase, MD 20815 USA; 7 Hospital for Dermato-Venereology, Ho Chi Minh City, Vietnam

**Keywords:** Diseases, Genetics, Neurology, Neuroscience

## Abstract

Leprosy is an infectious disease of the skin and peripheral nervous system. Sudden episodes of hyperinflammation, known as Type 1 Reactions (T1R), are a main contributor to permanent nerve damage in leprosy. The genetic component associated with the neuro-inflammatory phenotype of T1R displays pleiotropic effects with Parkinson’s disease (PD). In this study, we explored the genetic overlap between PD and T1R and expanded the evaluation of pleiotropic effects between T1R and other neurodegenerative disorders. We replicated the association of PD-linked rare variants in *PRKN* with T1R in Vietnamese leprosy patients. Analysis of 24 PD associated-genes revealed compound effects between rare protein-altering variants and T1R in the interacting genes *PRKN/PINK1* (*P* = 2.7^–05^; OR = 4.0) and a combination of rare/low frequency variants in the *LRRK2/GAK* pair (*P* = 6.7^–05^; OR = 0.54). These findings validated a genetic overlap between T1R and PD with two distinct axes, one of shared risk via *PRKN/PINK1* and a second of antagonistic pleiotropic via *LRRK2/GAK*. When testing an additional 94 genes associated with neurodegenerative diseases we identified variants in the amyotrophic lateral sclerosis disease-linked gene *TBK1* associated with T1R (*P* = 0.004; OR = 12.9). Our results highlight shared biological processes between leprosy and neurodegenerative diseases, which may indicate candidate drugs for repurposing to improve T1R management.

**Supplementary Information:**

The online version contains supplementary material available at 10.1038/s41598-025-30734-7.

## Introduction

Leprosy is a curable infectious disease caused by *Mycobacterium leprae* that primarily affects the skin and peripheral nervous system (PNS). Macrophages and Schwann cells, glia that form the myelin sheath in the PNS, are the main host cells of *M. leprae* (1). During the course of leprosy, 30% to 50% of patients experience episodes of hyperinflammation named type-1 reactions (T1R) (2). T1R are characterized by pathological cellular immune responses directed against the sites of infection. If not promptly treated, T1R is a major cause of permanent nerve impairment leading to irreversible damage to the neurons of the PNS. The recommended therapeutical intervention for T1R episodes is prolonged use of corticosteroids which carries significant adverse effects. Hence, the identification of prophylactic measures as well as novel and more tolerable therapies for T1R management remains of major public health priority in leprosy endemic areas in India, Brazil, and Indonesia*.*

We previously established a genetic component that predisposes leprosy patients to T1R (3–5). Through candidate gene approaches and genome-wide association studies, we identified regulatory variants in the *lncRNA ENSG00000235140,* in *TNFSF8,* and *TNFSF15,* and protein-altering variants in *LRRK2* and *PRKN* associated with T1R (3–7). A subset of these genetic factors exhibited pleiotropic effects between T1R and Parkinson’s Disease (PD) (6, 7). Identifying genes and their mutated domains that mediate pleiotropic effects can pinpoint optimal targets for therapeutic intervention and streamline drug repurposing, which represents an important advantage given the lengthy and costly nature of new drug development. To deepen our understanding of these pleiotropic mechanisms we employed a stepwise testing approach. First, we focused on the replication of the association of PD-linked genes *PRKN* and *LRRK2* with T1R in Vietnamese leprosy patients. Next, we evaluated if pleiotropic effects extended to other well-established PD-linked genes. Finally, we investigated if T1R shares genetic risk factors with other neurodegenerative diseases (e.g., Alzheimer’s disease, amyotrophic lateral sclerosis, dementia, and dystonia).

We replicated the association of rare protein-altering variants in *PRKN* with T1R in Vietnam. Additionally, we identified two distinct axes of pleiotropic effects between T1R and neurodegenerative disease linked genes: one axis of shared risk factors via *PRKN*, *PINK1*, *ADORA1,* and *TBK1* involved in mitochondrial homeostasis, cellular stress, and host response to intracellular bacteria, and a second axis of antagonistic pleiotropy via *LRRK2* and *GAK*.

## Results

### Protein-altering variants of neurodegenerative disease linked genes found among leprosy patients

**Fig. 1:**
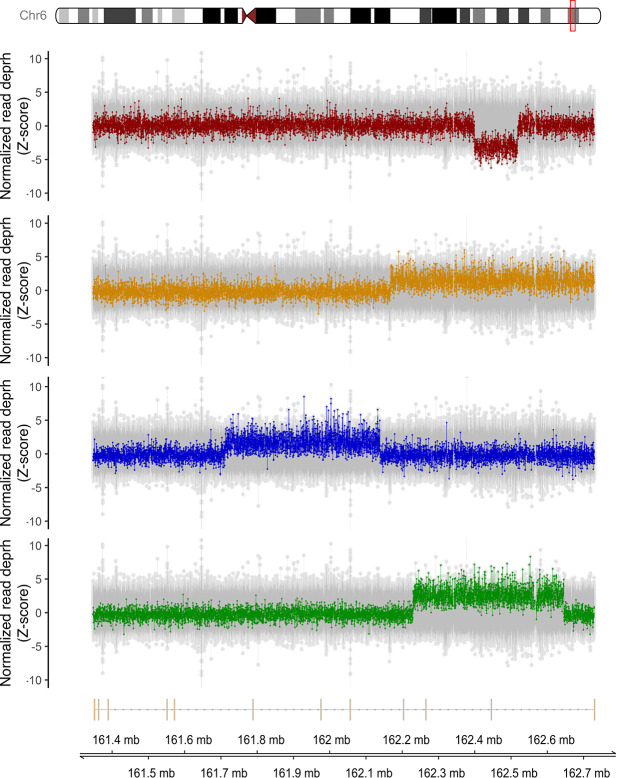
Structural variants in *PRKN* detected in T1R-affected participants. The four panels display the Z-scaled and PCA normalized depth of coverage on the y-axes across the *PRKN* locus shown on the x-axis. Each dot represents the depth of coverage for a probe at a specific chromosomal position within the locus. The coverage for the SV-presenting samples in each plot is highlighted in colors with the remaining 821 samples analyzed for structural variants shown in grey for comparison. *PRKN* structure is shown at the bottom with vertical lines correspond to exons.

For this study we assembled 412 T1R-affected and 419 T1R-free Vietnamese leprosy patients matched by sex, age, and distribution of leprosy clinical forms (Table 1). Of these, 430 individuals had been evaluated in our previous study reporting pleiotropic effects of *PRKN* and *LRRK2* between T1R and PD, while 403 were newly included participants. For all subjects, we sequenced the complete loci of 118 curated neurodegenerative disease associated genes included in Illumina’s TruSeq Neurodegeneration Panel. Of these genes, 24 have established association with PD (Fig. S1). We then performed variant calling and filtering for protein-altering and structural variants (SVs), which were subsequently tested for association with T1R. In total, we detected 1,223 protein-altering variants across 105 genes, while 13 genes had no protein variants among the study participants (Table S1). Twelve of the 1,223 variants were large SVs of which eleven were found in PD-linked genes, including four variants longer than 100 kilobases in *PRKN* (Table S1 and Fig. 1).

**Table 1:** Population phenotypic characteristics.

	T1R-free	T1R-affected
*Sex*		
Male	293	295
Female	119	124
*R&J classification*		
BT	92	92
BB	173	176
BL	148	151
*Age*		
Mean (SD)	23.56 (8.1)	22.83 (9.2)
Min – Max	5 – 67	5 – 66

BT, borderline tuberculoid; BB, borderline leprosy; BL, borderline lepromatous; R&J, Ridley and Jopling classification of leprosy clinical forms; SD, Standard deviation; T1R, Leprosy Type-1 reaction.

### Replication of pleiotropic effects for PRKN between T1R and PD.

**Fig. 2:**
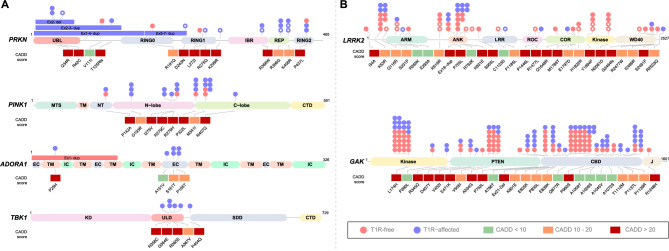
Protein-altering variants across the two axes of pleiotropic effects linking T1R and neurodegeneration-linked genes. For each gene, dots represent alleles of protein-altering variants with minor allele frequency < 1%. For GAK, three low-frequency variants associated with T1R protection are also shown. White dots in PRKN and LRRK2 mark alleles reported in our previous study (ref. 7). Bars indicate long structural variants (SVs). CADD score brackets below each gene schematic denote the predicted deleteriousness of each amino acid change and short SV. (A) Variants enriched in T1R-affected individuals in the T1R–Early-Onset Parkinson’s disease (EOPD) and T1R-Amyotrophic lateral sclerosis (ALS) pleiotropic axis. (B) Variants enriched in T1R-free individuals in the Late-Onset Parkinson’s disease (LOPD) antagonistic axis. UBL, UBiquitin-Like domain; RING Really Interesting New Gene finger domains, IBR, In-Between RING domain; REP, Repressor Element of Parkin; MTS, Mitochondrial Targeting Sequence domain; TM, Transmembrane domain; NT, N-Terminal tail domain; N-lobe and C-lobe of the Kinase domain; CTD, C-Terminal domain; KD, Kinase Domain; ULD, Ubiquitin-Like Domain; SDD, Scaffold Dimerization Domain; EC, Extracellular domain; IC, Intracellular domain. ARM, Armadillo repeats; ANK, Ankyrin repeats; LRR, Leucine-rich Repeats; ROC, Ras of Complex Proteins; COR, C-terminal of ROC. CBD, Clathrin-binding region.

We first tested if the burden of rare protein-altering variants (defined as minor allele frequency [MAF] < 1%) in *PRKN* and *LRRK2* differed between the 203 T1R-affected vs 200 T1R-free leprosy patients that had not been included in the previous study. We observed a significant accumulation of protein-altering variants for the *PRKN* gene in the T1R-affected cases as a direct replication of initial findings (Table 2). In the new T1R sample set we detected four rare heterozygote SVs, three resulted in multiple *PRKN* exons duplication and one in exon 2 deletion (Fig. 1). We also detected one *PRKN* frameshift (T125Rfs). These variants likely result in pronounced changes on Parkin structure and stability and were all found in T1R-affected cases (Fig. 2). We also detected a depletion of protein-altering variants for *LRRK2* in T1R-affected compared to T1R-free participants as we reported previously, however, this did not reach statistical significance (Table 2).

**Table 2:** Replication of accumulation of rare protein-altering variants in the *PRKN* and *LRRK2* genes in T1R patients.

Gene	T1R-affected	T1R-free	Cases/Controls	*P* value	OR (95% CI)	Samples
*PRKN*	1.9%	0.0%	216/214	0.003	7.61 (1.97—29.4)	Ref. *(7)*
	3.2%	1.0%	204/200	0.04	3.13 (1.04—9.37)	New
*LRRK2*	2.8%	6.3%	216/214	0.01	0.43 (0.23—0.82)	Ref. *(7)*
	2.7%	4.3%	204/200	0.36	0.69 (0.31—1.54)	New

Rare was defined as minor allele frequency < 1%; T1R, Leprosy Type-1 reaction. OR, odds ratio; CI, confidence interval.

### Additional PD-linked genes show pleiotropic effects with leprosy T1R

Next, we combined the 833 participants in a single population set and compared the burden of protein-altering variants between T1R-affected vs T1R-free participants for *PRKN*, *LRRK2*, and 22 additional PD-linked genes included in the neurodegenerative disease panel. We then performed a global analysis to estimate *PRKN* and *LRRK2* effect sizes in T1R susceptibility by combining the sample sets of the previous study with the current one (Table 3). Of note, we did not observe within gene compound heterozygotes for *PRKN*. In addition, we tested if T1R-affected cases or T1R-free controls carried different burden of protein-altering variants in 22 additional PD-linked genes that were not assessed in our previous study (Table S2). Three additional PD-associated genes (*PINK1, ADORA1* and *GAK*) showed significant differential burden of protein-altering variants between cases and controls at false discovery rate (FDR) < 5%. Rare protein-altering variants in *PINK1* and *ADORA1* were more frequent in T1R-affected cases while for GAK a protection effect was significant only when low frequency variants (MAF < 5%) were included (Table 3 and Fig. 2). For the *ADORA1* gene, we detected three variants clustered within the central extracellular domain, all found exclusively in T1R-affected cases. Given the small number of rare protein-altering allele counts identified in *ADORA1*, the estimated penetrance is imprecise and should be regarded as preliminary until validated in larger cohorts.

**Table 3:** Genewise association of rare variants with T1R in 833 participants.

Gene	Disease	MAF	Variants (count)	T1R affected	T1R free	*P value *^*a*^	*FDR* *P value *^*b*^	OR (95% CI)
*GAK*	PD	< 1%	22	3.8%	3.7%	0.44	0.73	0.80 (0.45 – 1.42)
*GAK*	PD	< 5%	25	5.9%	8.5%	0.004	0.03	0.53 (0.35 – 0.82)
*LRRK2*	PD	< 1%	27	2.2%	5.1%	0.001	0.02	0.39 (0.22 – 0.70)
*LRRK2*	PD	< 5%	29	5.5%	9.2%	0.006	0.03	0.55 (0.37 – 0.84)
*ADORA1*	PD	< 1%	5	0.7%	0.1%	0.007	0.03	10.0 (1.89 – 53.4)
*PINK1*	PD	< 1%	8	2.0%	0.6%	0.002	0.02	4.30 (1.68 – 11.0)
*PRKN*	PD	< 1%	17	2.4%	0.5%	0.003	0.02	3.77 (1.54 – 9.23)
*TBK1*	ALS	< 1%	5	0.7%	0.0%	0.004	0.05	12.9 (2.22 – 75.1)
*GAK – LRRK2*		< 5%	54	11.4%	17.7%	6.7E^-5^		0.54 (0.40 – 0.73)
*PRKN – PINK1*		< 1%	25	4.4%	1.1%	2.7E^-5^		4.01 (2–10 – 7.68)
*PRKN – PINK1 – TBK1*		< 1%	30	5.1%	1.1%	8.6E^-7^		4.61 (2.51 – 8.49)
*PRKN – PINK1 – TBK1 – ADORA1*		< 1%	35	5.8%	1.2%	2.8E^-8^		5.05 (2.85 – 8.96)

^*a*^* P* values estimated with the inverse of variance meta-analysis. ^b^ Benjamini–Hochberg false discovery rate (FDR) *P* values. T1R, Leprosy Type-1 reaction. T1R-affected (N = 420). T1R-free (N = 414). OR, odds ratio; CI, confidence interval; PD, Parkinson’s disease; ALS, Amyotrophic lateral sclerosis; MAF, Minor allele frequency

The proteins encoded by *PINK1* and *PRKN,* as well as those encoded by *LRRK2* and *GAK,* form two interaction pairs. Hence, we hypothesized that protein altering variants in the corresponding gene pairs may have additive effects by affecting the same molecular signalling pathways. We tested if the combined burden in each pair differed significantly between T1R-affected and T1R-free subjects. We observed a strong association of rare protein-altering variants in *PRKN* – *PINK1* with T1R-affected (Table 3). The burden of protein-altering variants in *GAK* – *LRRK2,* which was associated with protection from T1R, was primarily driven by independent variants (LD *r*^2^ = 0) within the 1% – 5% MAF range. These included three nonsynonymous variants in *GAK* (L174H, A534G, and Q877R), and the R1628P variant in *LRRK2* (Table 4). Notably, the association of *LRRK2* with T1R-free remained significant even in the absence of the low frequency R1628P variant (Table 3). This effect was mainly driven by *LRRK2* rare variants located in the critical ROC/COR and kinase domains and was particularly noticeable for the missense Y1894F and N2081D variants in the kinase domain, which were observed only in T1R-free participants (Fig. 2). Taken together, our results confirmed and expanded the existence of two axes of opposing pleiotropic effects between T1R and PD: the *PRKN/PINK1* axis, which predisposes to T1R, and the *LRRK2/GAK* axis, which confers T1R protection while both axes confer risk to PD.

**Table 4:** Low frequency protein-altering variants in *GAK* and *LRRK2*.

Gene	Variants^a^	T1R affected	T1R free	N	*P* *value*	OR (95% CI)	CADD Score	gnomAD	
								Global	EAS
*GAK*	Q877R	0.96%	2.91%	416/413	0.009	0.36 (0.16 – 0.77)	3.99	0.03%	0.77%
	A534G	0.51%	1.79%	390/390	0.004	0.22 (0.08 – 0.62)	25.2	0.003%	0.08%
	L174H	1.34%	2.31%	410/411	0.02	0.40 (0.18 – 0.88)	26.8	0.003%	0.01%
*LRRK2*	R1628P	2.80%	4.09%	393/379	0.12	0.63 (0.34 – 1.13)	27.5	0.09%	0.84%
	G2385R	1.20%	0.97%	374/361	0.54	1.39 (0.49 – 3.90)	23.2	0.08%	2.66%

^a^ Linkage disequilibrium (LD) *r*^2^ = 0 for the three *GAK* and the two *LRRK2* variants. T1R, leprosy Type-1 reaction; N, number of T1R-affected by T1R-free participants; OR, odds ratio; CI, confidence interval; CADD, Combined Annotation-Dependent Depletion; EAS, East Asian populations of gnomAD

### Pleotropic effects for T1R and neurodegenerative disease linked genes

In addition to PD, the TruSeq Neurodegeneration Panel includes 94 genes curated as either causal or strong risk factors for Alzheimer’s disease (AD), amyotrophic lateral sclerosis (ALS), frontotemporal dementia (FTD), dystonia, and other rare neurodegenerative conditions such as Huntington’s disease and Cerebellar Ataxia (Fig S1). We tested if any of these genes were associated with T1R (Table S2). We found protein-altering variants in the ALS-linked gene *TBK1* associated with susceptibility to T1R at FDR of 5% (Table 3). All *TBK1* variants were observed in T1R-affected cases and mainly located in or near the ubiquitin-like domain, a region critical for TBK1 activation and protein–protein interactions (Fig. 2). Similar to *ADORA1*, the small number of allele counts identified in *TBK1* inflates the odds ratio estimates, which should be interpreted with caution until the association with T1R is validated. No other gene surpassed the suggestive FDR < 10% in the studied population (Table S2).

### Genes of mitochondrial checkpoint are risk factors for T1R and neurodegenerative diseases

Protein altering variants in *TBK1* are most commonly associated with ALS and FTD. While parkinsonism syndrome is often observed in FTD cases, there is no substantial evidence that *TBK1* variants are associated with PD. However, *TBK1,* together with *PRKN* and *PINK1,* plays a critical role in mitochondrial quality control. Therefore, we hypothesized that genes involved in mitochondrial checkpoints represent a biological mechanism underlying pleiotropy between T1R and neurodegenerative diseases. We performed a meta-analysis combining the effects of *PRKN*, *PINK1*, and *TBK1*, and observed a more significant association with T1R (Table 3). We then expanded the meta-analysis to include *ADORA1*, a regulator of cellular stress that contributes to mitochondrial stability. The combined analysis of these four genes revealed a high effect size, suggesting a substantial contribution to T1R susceptibility in the Vietnamese population (*P* = 2.8E^-8^; OR = 5.05; confidence interval 95% (2.85 – 8.96)).

## Discussion

**Fig. 3:**
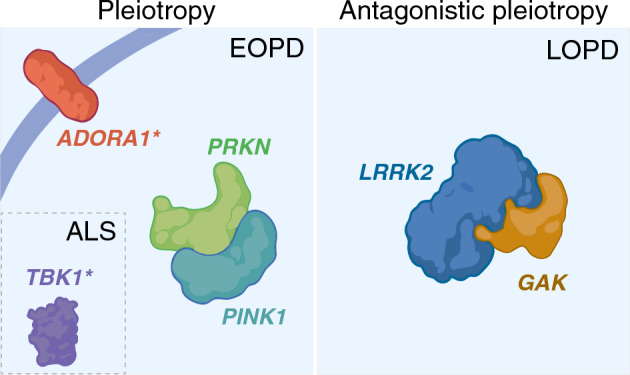
Two axes of pleiotropy between leprosy T1R and neurodegenerative diseases. The left panel shows pleiotropic associations between leprosy Type-1 reactions (T1R) and early-onset Parkinson’s disease (EOPD) as well as amyotrophic lateral sclerosis (ALS). The panel on the right shows antagonistic pleiotropic effects between T1R and late-onset Parkinson’s disease (LOPD). *Candidate genes.

We identified two distinct axes of pleiotropic effects between leprosy T1R and PD: one involving *LRRK2* and *GAK,* and another comprising the *PRKN* and *PINK1*. *LRRK2* and *GAK* are associated with both sporadic and familial forms of late-onset PD (LOPD**; **Fig. 3) (8). Nonsynonymous variants in *LRRK2* resulting in amino acid substitutions at positions 2019 and 1441 in the ROC/COR and kinase domains are known to cause LOPD, while regulatory variants of *GAK* have been shown to modulate PD risk across multiple ethnicities (9, 10). Conversely, autosomal recessive mutations in *PRKN* and *PINK1* are the two most prevalent causes of early-onset PD (EOPD**; **Fig. 3). The differences in age of onset and inheritance patterns between these two axes suggest that they may influence distinct aspects of PD pathogenesis. Our findings support the existence of two distinct mechanisms as LOPD-linked genes were associated with T1R-protection (i.e., antagonistic pleiotropy), while the EOPD-linked axis showed pleiotropy for both PD and T1R (Fig. 3).

In the antagonistic axis, LRRK2 is a multifunctional kinase that regulates various cellular processes, while GAK plays a crucial role in clathrin-mediated vesicle trafficking (10). LRRK2 and GAK function as interaction partners in a complex facilitated by BCL2-associated athanogene 5 (BAG5) (11). BAG5-mediated interaction occurs via the COR and kinase domains of LRRK2, the regions where the protein altering variant burden differed most significantly between T1R-free and T1R-affected individuals, and via the PTEN domain of GAK, which harbors the T1R-protective variant A534G. The LRRK2/GAK complex promotes clearance of Golgi-derived vesicles through autophagy (11). In the context of PD, defects in vesicle trafficking and clearance are hallmarks of α-synuclein accumulation in the brain. In leprosy and T1R, mycobacteria use the host’s autophagy machinery to evade the immune system and survive. However, the mechanism by which LRRK2 variants modulate this process deserves further investigation. For example, LRRK2/GAK protein altering variants could result in gain of function, as we have demonstrated for reactive oxygen species (ROS) production in the LRRK2 R1628P variant (7), which may facilitate vesicle clearance during the early stages of infection and prevent hyperactivation of the host immune system.

With respect to risk pleiotropic effects, we replicated our prior findings of rare protein-altering variants in *PRKN* as T1R risk factors (7). In addition, we detected the same large SVs observed in cases of EOPD in T1R-affected participants (12). Homozygous SVs or compound heterozygous SVs in combination with *PRKN* protein-altering variants represent up to 72% of *PRKN* mutations in European and Chinese EOPD cases (12–15). In EOPD, SVs occur most frequently in *PRKN* exons 2, 3, and 7, similar to what we observed in T1R-affected cases. However, in the heterozygous state and in the absence of additional mutations in other EOPD-linked genes, *PRKN* SVs are not associated with an increased risk of PD (15, 16). This difference in dominance effect between T1R and PD is consistent with the lack of epidemiological evidence suggesting a higher incidence of PD in T1R or leprosy affected individuals. Despite this, healthy carriers of *PRKN* heterozygote protein altering variants have shown increased serum levels of pro-inflammatory cytokines (e.g., IL-6, IL-1β, CCL2) similar to those observed in homozygote *PRKN* protein altering variant carriers with PD (17). This suggests that *PRKN* modulates the extent of the immune response irrespective of the T1R or PD phenotype. Moreover, in a mouse model the pro-inflammatory state associated with loss of *PRKN* activity was STING-mediated (17). This provides a direct link to *TBK1*, a critical downstream effector in STING signaling (17). In our study, we only observed protein altering variants in the ubiquitin-like domain of *TBK1* and only in T1R participants. STING –TBK1 play a pivotal role in autophagy, antimicrobial defence, and antigen presentation (18, 19). Deletion of the UBL domain was shown to impair TBK1 kinase activity (20) which plays a key role in autophagosome formation and pro-inflammatory signalling (18, 19). The key role of *PRKN* signalling in both T1R and PD was further highlighted by our finding of an enrichment of *PINK1* protein altering variants in T1R patients. The most frequent *PINK1* variants associated with EOPD are nonsynonymous variants located in the kinase domain. A similar pattern was observed in T1R, where the vast majority of *PINK1* protein altering variants clustered in the C- and N-lobe of the PINK1 kinase domain. Phosphorylation of Parkin by PINK1 is required to activate its autoinhibited Parkin ubiquitin ligase domain (21). The functional role of *PRKN/PINK1* activity is among the best characterized mechanisms in the pathogenesis of EOPD.

The interplay between PINK1 and Parkin is crucial for selective autophagy and immune response processes, such as mitophagy, mitochondrial antigen presentation via mitochondrial-derived vesicles (MitAP), and regulation of adaptive immunity. These biological processes may represent the key components underlying the pleiotropic effect between T1R and PD. In mitophagy, PINK1 and Parkin mark damaged mitochondria for degradation through ubiquitination. Failure to remove damaged mitochondria leads to oxidative stress via increase in intracellular production of ROS (22). Stimulation of adenosine receptors, including the T1R-risk gene *ADORA1*, protect against oxidative damage during neuroinflammation (23, 24). Therefore, defects in mitochondrial homeostasis and cellular stress might be linked to inflammation in both PD and T1R. In xenophagy, a host response mechanism against intracellular pathogens, PINK1 and Parkin ubiquitinate phagosomes containing intracellular pathogens. However, certain bacteria, such as *M. tuberculosis*, escape xenophagy, survive and replicate in *PRKN* knockout macrophages *(25, 26)*. Defects in PINK1/Parkin-mediated mycobacterial clearance could explain the previously observed association between *PINK1* and *PRKN* with leprosy per se (27–29). In T1R, the contribution of xenophagy had dichotomous findings with T1R episodes linked to blockade and activation (30). Therefore, alternative mechanisms such as MitAP are candidates for the underlying biological role of *PRKN/PINK1* in T1R. In MitAP, PINK1 and Parkin suppress mitochondrial antigen presentation to immune cells (31). Loss of *PRKN* and *PINK1* function impairs this suppression, which could contribute to the immune mediated neurodegeneration in PD and the excessive inflammation observed in T1R (17). In PD, MitAP functions independently of autophagy; however, the extent to which this applies to T1R remains unknown.

The results for *GAK/LRRK2* and *PINK1/PRKN* replicate and extend our previous findings implicating LRRK2 and Parkin in T1R pathology (7). Collectively, these data indicate that the detected amino acid substitutions influence protein function and contribute to T1R susceptibility. In contrast, the associations of rare amino acid changes in TBK1 and ADORA1 with T1R are novel and should be regarded as hypothesis-generating. The T1R-associated variants in the ubiquitin-like domain of *TBK1* are likely to alter kinase activity, potentially modulating autophagy via the TBK1-optineurin loop (32) or apoptosis through the IRF3/Bax pathway (33). Variants in *ADORA1* associated with increased T1R risk may also promote apoptosis, as inhibition of *ADORA1* activity is a potent inducer of this process. Both autophagy and apoptosis have previously been implicated in T1R episodes (34, 35). Together, these findings support a model in which hemizygous amino acid substitutions in TBK1 and ADORA1 reduce protein activity and modulate cellular pathways contributing to T1R pathology.

The dynamics of the immune response are controlled by a complex interplay between genomic and environmental factors, where individual genes often regulate multiple aspects of disease pathogenesis. To illustrate this, excessive inflammation increases the risk of tissue damage while insufficient inflammation predisposes a person to infections or facilitates pathogen dissemination. Thus, maintaining a balance between protective and damaging responses is essential for immunological homeostasis. A compelling example is the *LRRK2* R1628P variant, which has been associated with T1R-protection when comparing T1R-affected and T1R-free leprosy patients (7). However, when each T1R subgroup (affected and unaffected) was compared independently to healthy controls, the R1628P variant showed an opposing effect (36). The 1628P allele was protective in the T1R-affected subgroup but associated with susceptibility to leprosy per se in the T1R-free group. Interestingly, the association of R1628P with leprosy per se was also observed in the Chinese population (37). This seemingly paradoxical observation exemplifies how a single variant can shape the outcome of leprosy and its endophenotypes.

The identification of pleiotropic biological processes offers valuable opportunities for therapeutic repurposing. This strategy is particularly important for neglected tropical diseases such as leprosy and T1R, where research and development resources are limited. In contrast, several drugs and compounds targeting PD-linked genes including *PRKN*, *PINK1*, GAK and *LRRK2* are currently under active investigation or enrolled in clinical trials for PD (38, 39). Although standard PD treatments such as dopamine-targeting drugs (e.g., levodopa), are unlikely to be relevant for leprosy or T1R, compounds that modulate pleiotropic genes may be promising candidates for repurposing in leprosy/T1R. For example, the allosteric modulator tetrahydropyrazolo-pyrazine (THPP) functions as a molecular glue that enhances the activity of Parkin’s UBL domain (40). THPP has been shown to partially rescue Parkin UBL activity in EOPD cases carrying R42P variant, the same residue observed mutated in T1R-affected subjects and affected by SVs. However, the rescue effect of THPP is limited to the ubiquitin function of Parkin, which may constrain its therapeutic relevance for T1R where additional protein altering variants in different domains may impair other Parkin functions or interactions.

Another promising candidate for repurposing is the small molecule Kinetin riboside, which stimulates PINK1-dependent mitophagy (41, 42). In parallel, additional patents have been filed for small molecules also targeting PINK1 and Parkin-mediated mitophagy (43). However, several of these molecules showed limited effects in PD rodent models and will requiring further optimization and development (19, 38). An alternative strategy currently being explored in PD involves inhibiting Parkin deubiquitinating enzymes (44). For instance, a USP30 knockout has been shown to rescue mitophagy defects caused by pathogenic Parkin amino acid changes. Compounds targeting ubiquitin-specific proteases, including USP30, and other deubiquitinases such as ataxin-3 are under clinical investigation (45, 46). Among the most advanced candidates are substituted cyanopyrrolidines (44). Molecules targeting the T1R antagonistic pleiotropic genes *LRRK2* and *GAK* are also under investigation in PD (38). However, due to the variant-dependent effects of LRRK2 in leprosy and its associated endophenotypes, a more tailored selection of candidate compound will be necessary. While many of these compounds are not yet clinically available, partnerships between academic researchers and the commercial developers could provide valuable insights into their broader application. In this context, our genetic findings represent an important first step, highlighting key contributors within candidate pathways implicated in the pathophysiological mechanisms of T1R. Testing compounds targeting T1R-risk genes in vitro, particularly in the context of the identified mutated domains, would be a logical next step and could help guide therapeutic interventions and human trials. Such collaborations may facilitate future repurposing of both existing and novel therapies for leprosy patients with T1R, who currently rely heavily on long-term corticosteroid treatments.

Our study has some limitations. Although cases and controls were matched by age, sex at birth, and clinical form of leprosy by design, potential unknown residual confounders (e.g., bacterial load, number of reactional episodes, and drug therapy) could contribute the association of Neurodegenerative-linked genes with T1R. Moreover, although we controlled for false positives by validating singletons with independent deep sequencing, low coverage may have reduced sensitivity to detecting rare variants. This limitation would affect both cases and controls equally, as sequencing depth did not differ between groups. Additionally, the sample size of our study was relatively small and may have resulted in false negatives gene-wise associations due to limited statistical power. Future studies with larger and more diverse populations, particularly in leprosy endemic regions, will be important to validate and extend our pleiotropic findings.

In conclusion, we identified shared biological processes involving excessive inflammatory responses in leprosy with known PD-linked genes. Our findings convey two important messages: First, they reinforce the hypothesis of an inflammatory component in PD, potentially mediated by an infectious agent, as proposed in a brain-gut axis model. Second, they highlight that excessive inflammation with resulting neuronal damage, in the PNS for leprosy and CNS for PD, is mediated by a shared set of genes that serve as key checkpoints in immune-mediated processes.

## Matherials and methods

### Population sample and targeted sequencing of neurodegenerative diseases linked genes

Written informed consent was obtained from all participants or from parent/guardian of minors. The study was approved by the Research Ethics Board at the Research Institute at McGill University Health Centre in Montreal (REC98-041) and the regulatory authorities of Ho Chi Minh City in Vietnam (So3813/UB-VX and 4933/UBND-VX). All research was performed in accordance with relevant guidelines/regulations and in accordance with the Declaration of Helsinki. We retrieved DNA from 940 leprosy cases from our biobanked samples of the Vietnamese population. Of these, 470 were leprosy cases presenting at least one T1R episode, and 470 were T1R-free leprosy patients absent of any type of leprosy reactions (e.g., Erythema Nodosum Leprosy and neuritis) matched by sex, age of onset and clinical form of the disease. A DNA aliquot of 50 ng/uL from each participant was then used to target sequence PD-linked genes with the TruSeq Neurodegeneration Panel. This panel was designed by Illumina with input of neurodegenerative researchers to target sequence the entire locus of 118 selected genes (~ 8.6 mega bases). In addition to PD-linked genes, the panel covers a broad spectrum of neurodegenerative conditions such as Alzheimer’s disease, ALS, Frontotemporal Dementia among other rare neurodegenerative disorders (Fig S1). Libraries were prepared according to Illumina manufacture instructions. TrueSeq libraries from all participants were 150 bp paired-end sequenced with Illumina HiSeq 4000.

### Variant calling and quality control

Sequenced libraries were processed in the high-performance computational platform Cedar from the Digital Research Alliance of Canada. Nextera adaptors were trimmed using TrimGalore v0.4.5. Reads were aligned to the human genome hg38 build using BWA mem v.0.7.17 with default parameters and duplicate reads were marked with PICARD v.2.18.9. Subjects with mean depth of coverage < 10X or samples with relatedness > 0.1 were excluded from further analysis. Of the 940 samples 833 passed quality control. For these participants we obtained a mean depth of coverage of 20X per gene in the Neurodegenerative panel (Fig S2). Next, variants were called using HaplotypeCaller from GATK v.4.0.8.1 and DeepVariant v.1.0 in WES mode. Cohort genotyping of individual gVCFs was performed with GenotypeGVCFs from GATK and GLNexus for DeepVariant. Variants with Q < 30, Depth < 10X, and missing > 20% were excluded. Protein-altering variants with consensus calls from both pipelines were retained for further analysis. Singletons supported by less than 15 reads in associated genes were validated by deep sequencing using a custom panel from Twist Bioscience. These strategies were applied to minimize false-positive rare variant calls in loci with low coverage.

### Structural variants detection

The probes designed for the neurodegeneration panel captured continuous regions of the genome, including gene flanking regions, exons, introns, and promoters of the targeted genes. Hence, the depth of coverage of sequential probes can be used to estimate the occurrence of SVs. We used the probe location to estimate the depth of coverage by probe per sample to detect large SVs such as deletions, duplications and copy number variants (CNVs). Briefly, reads with Phred > 20 were used to estimate the depth per probe loci using GATK *DepthOfCoverage* for the 833 participants. We then used GATK *GCContentByInterval* to detect regions with extreme GC content and plinkseq v.0.10 to detect regions of low complexity. These regions could interfere with data normalization and were excluded from the SV calling. Next, XHMM was used to perform a principal component (PC) analysis with the normalized counted data. We then regressed out the top 19 principal components that cumulatively explained 70% of the total variance and Z-scaled the data per sample as per recommendation. To detect SVs, XHMM –discover and –genotype was used with default parameters and distance between targets of 500 bp. As quality control filtering, SVs with > 15 sequential probes deviating one standard deviation from the population mean Z-scaled coverage and encompassed exons were selected for analysis*.* Large deletions encompassing exonic regions in key genes were validated by whole genome sequencing. Due to limited power of detectability, short SVs reported in this study need to be interpreted with caution as they require further validation.

### Data analysis

The stepwise protein-altering variant analysis was performed with regenie v.3.6. First, ~ 19 thousand variants (coding and non-coding) with MAF > 1% detected in the 118 targeted genes was used to calculate a null model with –step 1 and –bt to define a binary phenotype. This first step was carried out to capture the genetic contributions to the phenotype while accounting for population structure and relatedness. Including age and sex as covariates in –step 1 had minimal impact on the association *P* values given the matched design of our study and the homogeneity of the Vietnamese Kinh population. Next, regenie –step 2 was used to estimate differences in rare variant burden between cases and controls under additive models or kernel models with –vc-tests skato-acat. The analysis used the prediction file generated in –step 1, a set list including 1,223 protein-altering variants split by their corresponding gene, and –rgc-gene-p to estimate one *P* value per gene in two MAF thresholds of < 1% and < 5%.

We carried out protein-altering variants testing in three stages: (i) split by the population described in Ref (7), and the newly included participants for the *PRKN* and *LRRK2* genes. We then compared different burden between cases – controls for (ii) PD-linked genes and (iii) genes associated with other neurodegenerative conditions. Genes with < 5 protein-altering variants were excluded from testing. As the gene-wise enrichment testing followed our stepwise design, the Benjamini–Hochberg FDR correction was calculated first for T1R vs PD and then for T1R vs other neurodegenerative diseases*.* To test the combined effect of the interactive proteins we performed an inverse of the variance meta-analysis using *β* and standard errors of the burden for each gene in R environment. The test of individual low frequency variants for *LRRK2* and *GAK* was carried out in a similar fashion as the genewise tests but without defining a set of variants per gene.

## Supplementary Information

Supplementary Information 1.

Supplementary Information 2.

## Data Availability

The data, code, protocols, and key lab materials used and generated in this study are listed in a Key Resource Table (KRT) in supplement. NGS data are not publicly available due to participant consent restrictions. NGS data are available for leprosy-related studies by requests to the Bioinformatics Platform at the Research Institute of the McGill University Health Centre (bioinformatics.rimuhc@mcgill.ca). Approval from the applicant’s ethics committee is required for additional work on the data. No personal identification of the studied family will be shared due to consent restrictions. All relevant data are within the manuscript and its Supporting Information files. An anonymized pipeline for data processing and analysis is available via the link in KRT.
