# Supplementary material for: Bridging pleiotropic mechanisms in leprosy type-1 reactions and neurodegenerative diseases
Source: Sci Rep. 2025 Dec 22;15:45036. doi: 10.1038/s41598-025-30734-7 (PMC12749272; doi:10.1038/s41598-025-30734-7)
Supplement: Supplementary file 1 — Supplementary Information 1. [file 41598_2025_30734_MOESM1_ESM.pdf]

Bridging Pleiotropic Mechanisms in Leprosy Type-1 Reactions and Neurodegenerative diseases

Vinicius M. Fava [1,2,3,*], Jônatas Perico [4,5], Marianna Orlova [1,2], Monica Dallmann-Sauer [1,2], Yong Zhong Xu [1,2,6], Nguyen Van Thuc [7], Vu Hong Thai [7], Andrea F. Belone [4], Ana Carla P Latini [4,5], and Erwin Schurr [1,2,6].

[1] Program in Infectious Diseases and Global Health, The Research Institute of the McGill University Health Centre, Montreal, Canada.

[2] McGill International TB Centre, Department of Medicine, Faculty of Medicine, McGill University, Montreal, Canada.

[3] Present address. Canadian Centre for Computational Genomics (C3G) McGill University, Montreal, Canada.

[4] Instituto Lauro de Souza Lima, Bauru, São Paulo, Brazil.

[5] Program in Tropical Diseases, São Paulo State University (Unesp), Medical School, Botucatu, São Paulo, Brazil.

[6] Aligning Science Across Parkinson's (ASAP) Collaborative Research Network, Chevy Chase, MD 20815.

[7] Hospital for Dermato-Venereology, Ho Chi Minh City, Vietnam

SUPPLEMENTARY MATERIAL

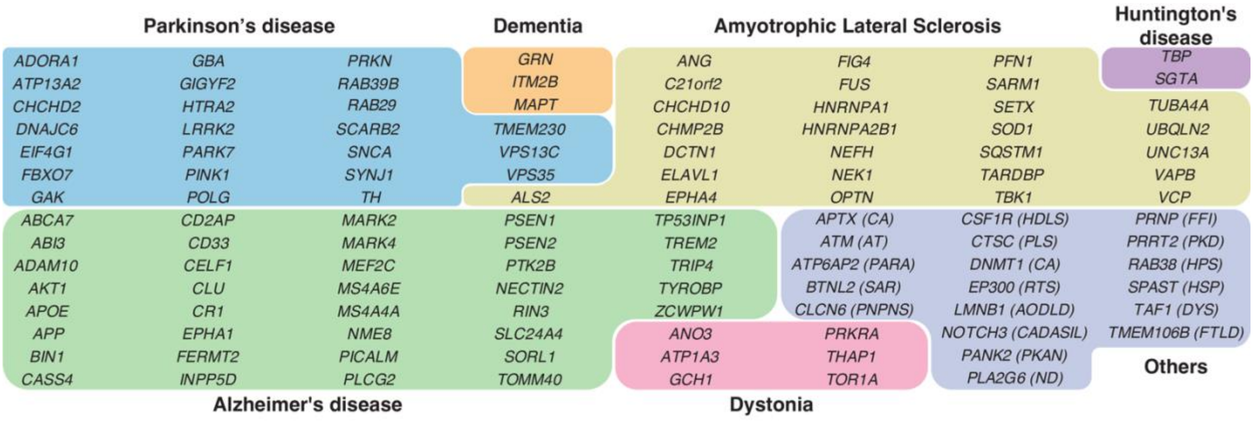

Fig S1. Most frequent phenotype associated with genes in the Neurodegeneration panel. The 118 genes sequenced in the Illumina TruSeq Neurodegeneration Panel® were clustered according to the most frequent phenotype impacted or associated with the gene. The "Others" category included: AODLD, Adult-Onset Leukodystrophy; AT, Ataxia Telangiectasia; CA, Cerebellar Ataxia; CADASIL, Cerebral Autosomal Dominant Arteriopathy with Subcortical Infarcts and Leukoencephalopathy; PNPNS, Childhood-onset progressive neurodegeneration-peripheral neuropathy syndrome; FFI, Fatal familial insomnia; FTLD, Frontotemporal lobar degeneration; HDLS, Hereditary diffuse leukoencephalopathy with spheroids; HSP, Hereditary Spastic Paraplegia; HPS, Hermansky-Pudlak syndrome; ND, Neuroaxonal dystrophy; PKAN, Pantothenate Kinase-Associated Neurodegeneration; PLS, Papillon-Lefèvre Syndrome; PKD, Paroxysmal Kinesigenic Dyskinesia; RTS, Rubinstein-Taybi syndrome; SAR, sarcoidosis; DYS, X-Linked Dystonia; PARA, X-Linked Paraganglioma.

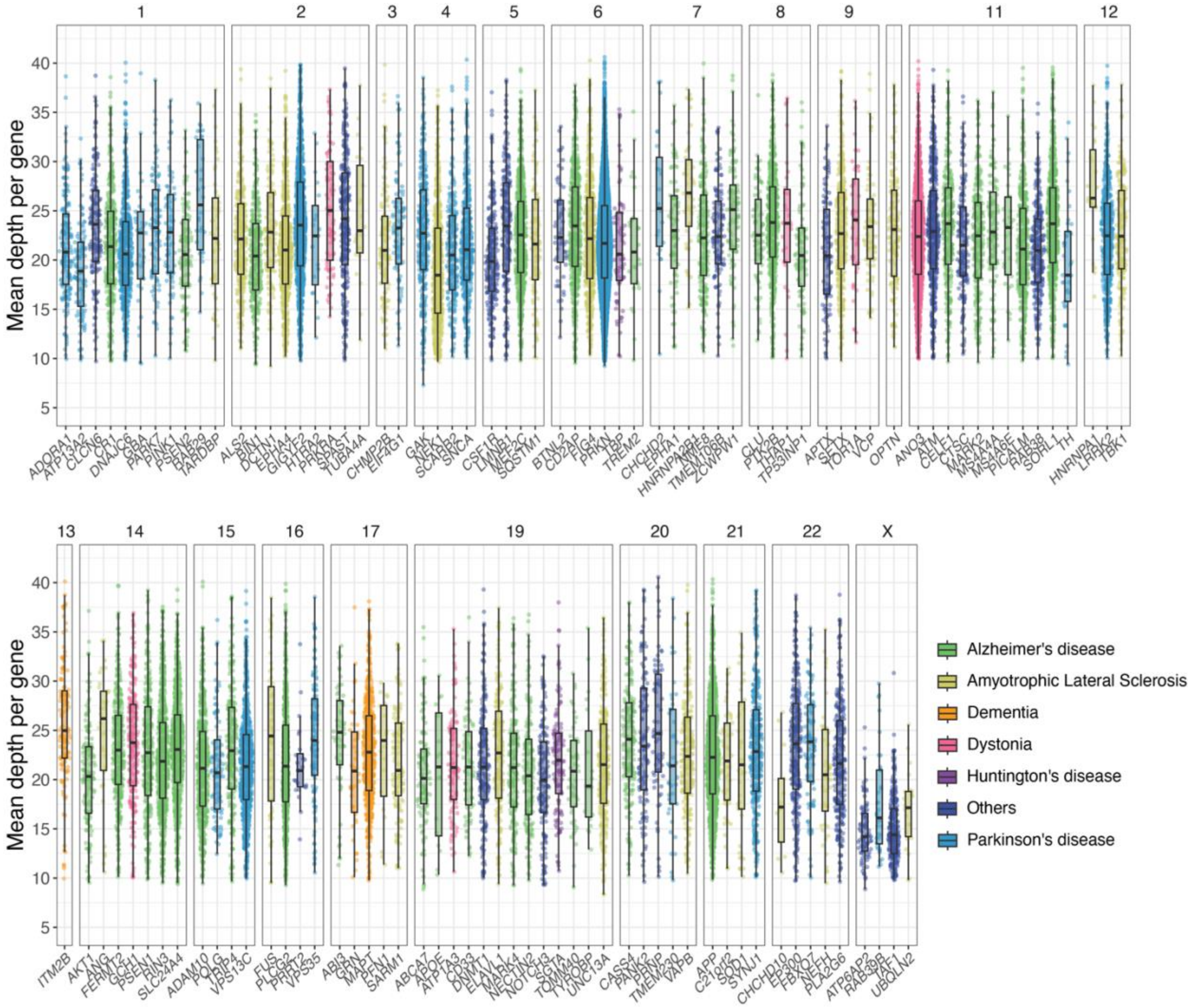

Fig S2. Mean sequencing coverage per gene of the Neurodegeneration panel.

Depth of coverage for genes in the Neurodegeneration panel among 876 leprosy-affected subjects. Each dot represents a probe capturing up to 300 base-pairs at the gene loci colored according to the most frequently associated neurological phenotype. The y-axis indicates the mean depth for each gene and the x-axis shows the respective gene (split by chromosome).

Table S2. Rare variant analysis in neurodegenerative diseases associated genes.

			MAF < 1%							MAF < 5%						
Gene	Disease	All Protein altering variants	T1R-affected (N = 420)	T1R-free (N = 414)	*Burden P value*	*FDR P value*	*β*	SE	OR (95% CI)	T1R-affected (N = 420)	T1R-free (N = 414)	*Burden P value*	*FDR P value*	*β*	SE	OR (95% CI)
ADORA1	PD	5	0.7%	0.1%	0.007	0.032	2.31	0.85	10.05 (1.89 - 53.39)	0.7%	0.1%	0.007	0.026	2.31	0.85	10.05 (1.89 - 53.39)
ATP13A2	PD	20	3.8%	3.5%	0.26	0.63	0.33	0.29	1.39 (0.78 - 2.47)	5.8%	4.7%	0.10	0.26	0.42	0.25	1.52 (0.93 - 2.5)
CHCHD2	PD	5	0.8%	0.5%	0.63	0.73	0.32	0.67	1.38 (0.37 - 5.14)	0.8%	0.5%	0.63	0.73	0.32	0.67	1.38 (0.37 - 5.14)
DNAJC6	PD	17	2.0%	1.8%	0.81	0.82	0.10	0.41	1.1 (0.5 - 2.44)	2.0%	1.8%	0.81	0.82	0.10	0.41	1.1 (0.5 - 2.44)
EIF4G1	PD	24	3.7%	4.6%	0.64	0.73	-0.13	0.28	0.88 (0.51 - 1.51)	5.8%	6.5%	0.52	0.73	-0.15	0.24	0.86 (0.54 - 1.36)
FBXO7	PD	9	0.6%	0.6%	0.57	0.73	0.40	0.71	1.49 (0.37 - 5.98)	0.6%	0.6%	0.57	0.73	0.40	0.71	1.49 (0.37 - 5.98)
GAK	PD	27	3.8%	3.7%	0.44	0.73	-0.23	0.29	0.8 (0.45 - 1.42)	5.9%	8.5%	0.004	0.026	-0.63	0.22	0.53 (0.35 - 0.82)
GBA	PD	8	1.3%	0.5%	0.18	0.50	0.78	0.59	2.19 (0.69 - 6.94)	1.3%	0.5%	0.18	0.44	0.78	0.59	2.19 (0.69 - 6.94)
GIGYF2	PD	29	3.1%	4.3%	0.12	0.39	-0.46	0.30	0.63 (0.35 - 1.13)	5.8%	7.2%	0.26	0.54	-0.27	0.23	0.77 (0.49 - 1.21)
HTRA2	PD	8	1.0%	1.0%	0.82	0.82	-0.14	0.60	0.87 (0.27 - 2.84)	1.0%	1.0%	0.82	0.82	-0.14	0.60	0.87 (0.27 - 2.84)
LRRK2	PD	33	2.2%	5.1%	0.001	0.023	-0.93	0.29	0.39 (0.22 - 0.7)	5.5%	9.2%	0.006	0.026	-0.59	0.21	0.55 (0.37 - 0.84)
PINK1	PD	10	2.0%	0.6%	0.002	0.023	1.46	0.48	4.3 (1.68 - 11.04)	2.0%	0.6%	0.002	0.026	1.46	0.48	4.3 (1.68 - 11.04)
POLG	PD	23	1.9%	3.5%	0.03	0.10	-0.75	0.34	0.47 (0.24 - 0.92)	6.8%	8.9%	0.08	0.24	-0.38	0.21	0.69 (0.45 - 1.04)
PRKN	PD	19	2.4%	0.5%	0.004	0.023	1.33	0.46	3.77 (1.54 - 9.23)	2.4%	0.5%	0.004	0.026	1.33	0.46	3.77 (1.54 - 9.23)
SCARB2	PD	12	1.6%	1.9%	0.57	0.73	-0.25	0.43	0.78 (0.33 - 1.83)	1.6%	1.9%	0.57	0.73	-0.25	0.43	0.78 (0.33 - 1.83)
SYNJ1	PD	28	2.3%	2.3%	0.55	0.73	0.22	0.37	1.25 (0.61 - 2.57)	2.3%	2.3%	0.55	0.73	0.22	0.37	1.25 (0.61 - 2.57)
TH	PD	18	1.9%	2.1%	0.65	0.73	-0.17	0.38	0.84 (0.4 - 1.78)	1.9%	2.1%	0.65	0.73	-0.17	0.38	0.84 (0.4 - 1.78)
VPS13C	PD	70	8.2%	8.2%	0.49	0.73	0.14	0.21	1.15 (0.77 - 1.74)	14.4%	14.4%	0.30	0.56	0.18	0.17	1.2 (0.86 - 1.67)
VPS35	PD	9	0.8%	0.7%	0.584	0.730	0.33	0.60	1.39 (0.43 - 4.54)	0.8%	0.7%	0.584	0.730	0.33	0.60	1.39 (0.43 - 4.54)
ABCA7	AD	59	7.0%	5.1%	0.07	0.59	0.42	0.24	1.53 (0.96 - 2.43)	13.5%	11.7%	0.21	0.61	0.22	0.18	1.25 (0.88 - 1.76)
ABI3	AD	12	1.1%	1.4%	0.37	0.76	-0.46	0.51	0.63 (0.23 - 1.72)	1.1%	1.4%	0.37	0.61	-0.46	0.51	0.63 (0.23 - 1.72)
APOE	AD	9	0.7%	0.5%	0.34	0.76	0.69	0.73	2 (0.48 - 8.31)	0.7%	0.5%	0.34	0.61	0.69	0.73	2 (0.48 - 8.31)
BIN1	AD	7	0.4%	0.6%	0.31	0.76	-0.84	0.84	0.43 (0.08 - 2.22)	3.4%	2.8%	0.54	0.72	0.21	0.33	1.23 (0.64 - 2.36)
CASS4	AD	12	1.2%	0.8%	0.66	0.85	0.24	0.55	1.27 (0.43 - 3.78)	2.6%	3.5%	0.28	0.61	-0.35	0.32	0.71 (0.38 - 1.33)
CD2AP	AD	8	1.2%	0.6%	0.43	0.80	0.46	0.59	1.58 (0.5 - 4.98)	1.2%	0.6%	0.434	0.651	0.46	0.59	1.58 (0.5 - 4.98)
CD33	AD	13	2.5%	2.5%	0.72	0.86	0.13	0.36	1.14 (0.57 - 2.28)	6.2%	6.4%	0.91	0.91	0.03	0.23	1.03 (0.66 - 1.6)
CLU	AD	9	0.6%	0.6%	0.62	0.85	0.36	0.72	1.43 (0.35 - 5.9)	0.6%	0.6%	0.62	0.78	0.36	0.72	1.43 (0.35 - 5.9)
CR1	AD	13	0.6%	0.7%	0.87	0.88	0.12	0.71	1.12 (0.28 - 4.5)	0.6%	0.7%	0.87	0.91	0.12	0.71	1.12 (0.28 - 4.5)
EPHA1	AD	15	0.6%	1.1%	0.596	0.849	-0.31	0.58	0.73 (0.23 - 2.3)	1.2%	2.4%	0.030	0.609	-0.88	0.41	0.41 (0.19 - 0.92)
FERMT2	AD	6	1.2%	0.8%	0.875	0.875	0.08	0.53	1.09 (0.39 - 3.06)	1.2%	0.8%	0.875	0.911	0.08	0.53	1.09 (0.39 - 3.06)
INPP5D	AD	11	1.3%	1.4%	0.77	0.88	-0.14	0.48	0.87 (0.34 - 2.23)	1.3%	1.4%	0.77	0.88	-0.14	0.48	0.87 (0.34 - 2.23)
NECTIN2	AD	8	0.4%	0.7%	0.171	0.683	-1.02	0.74	0.36 (0.08 - 1.55)	2.3%	3.0%	0.272	0.609	-0.38	0.35	0.68 (0.35 - 1.35)
NME8	AD	16	1.8%	2.3%	0.32	0.76	-0.38	0.38	0.69 (0.32 - 1.45)	1.8%	2.3%	0.32	0.61	-0.38	0.38	0.69 (0.32 - 1.45)
PICALM	AD	5	0.6%	0.7%	0.67	0.85	-0.28	0.64	0.76 (0.22 - 2.66)	0.6%	0.7%	0.67	0.80	-0.28	0.64	0.76 (0.22 - 2.66)
PLCG2	AD	12	1.3%	1.1%	0.67	0.85	0.21	0.49	1.23 (0.47 - 3.19)	2.9%	1.9%	0.09	0.61	0.60	0.36	1.82 (0.9 - 3.65)
PSEN2	AD	7	1.0%	0.5%	0.38	0.76	0.54	0.62	1.72 (0.51 - 5.82)	1.0%	0.5%	0.38	0.61	0.54	0.62	1.72 (0.51 - 5.82)
PTK2B	AD	18	1.9%	1.8%	0.831	0.875	0.09	0.40	1.09 (0.5 - 2.37)	3.2%	2.8%	0.527	0.717	0.20	0.32	1.22 (0.66 - 2.28)
RIN3	AD	25	3.1%	1.9%	0.12	0.59	0.54	0.35	1.72 (0.86 - 3.44)	8.5%	6.8%	0.34	0.61	0.20	0.21	1.22 (0.81 - 1.86)
SLC24A4	AD	13	0.4%	1.1%	0.08	0.59	-1.09	0.61	0.34 (0.1 - 1.12)	0.4%	1.1%	0.08	0.61	-1.09	0.61	0.34 (0.1 - 1.13)
SORL1	AD	29	4.8%	4.0%	0.53	0.85	0.17	0.27	1.19 (0.7 - 2.01)	6.6%	5.2%	0.33	0.61	0.23	0.23	1.26 (0.79 - 1.99)
TREM2	AD	9	1.6%	1.0%	0.31	0.76	0.46	0.45	1.58 (0.66 - 3.81)	2.5%	1.9%	0.36	0.61	0.33	0.35	1.38 (0.69 - 2.77)
TRIP4	AD	6	0.5%	1.3%	0.12	0.59	-0.84	0.54	0.43 (0.15 - 1.24)	0.5%	1.3%	0.12	0.61	-0.84	0.54	0.43 (0.15 - 1.24)
ZCWPW1	AD	11	1.6%	2.7%	0.12	0.59	-0.58	0.38	0.56 (0.27 - 1.17)	4.0%	4.8%	0.142	0.609	-0.40	0.27	0.67 (0.4 - 1.14)
ALS2	ALS	15	1.6%	1.8%	0.94	0.94	-0.03	0.44	0.97 (0.41 - 2.29)	4.4%	4.6%	0.88	0.96	-0.04	0.27	0.96 (0.57 - 1.63)
C21orf2	ALS	6	0.4%	0.4%	0.81	0.92	-0.24	0.97	0.79 (0.12 - 5.24)	7.4%	7.6%	0.85	0.96	0.04	0.20	1.04 (0.7 - 1.54)
DCTN1	ALS	14	1.1%	1.8%	0.14	0.57	-0.68	0.46	0.51 (0.21 - 1.25)	2.6%	4.2%	0.02	0.14	-0.70	0.31	0.5 (0.27 - 0.9)
FIG4	ALS	11	0.8%	1.7%	0.135	0.567	-0.71	0.48	0.49 (0.19 - 1.25)	0.8%	1.7%	0.135	0.567	-0.71	0.48	0.49 (0.19 - 1.25)
FUS	ALS	7	0.7%	0.2%	0.174	0.567	1.13	0.83	3.1 (0.61 - 15.88)	0.7%	0.2%	0.174	0.567	1.13	0.83	3.1 (0.61 - 15.88)
NEFH	ALS	23	3.0%	3.4%	0.43	0.70	-0.24	0.31	0.78 (0.43 - 1.44)	4.7%	4.5%	0.97	0.97	-0.01	0.27	0.99 (0.59 - 1.66)
NEK1	ALS	14	2.2%	1.6%	0.408	0.697	0.33	0.40	1.39 (0.64 - 3.04)	2.2%	1.6%	0.408	0.883	0.33	0.40	1.39 (0.64 - 3.04)
OPTN	ALS	8	0.7%	0.5%	0.57	0.82	0.39	0.68	1.48 (0.39 - 5.63)	3.5%	2.8%	0.79	0.96	0.09	0.32	1.09 (0.59 - 2.02)
SARM1	ALS	5	0.4%	0.6%	0.85	0.92	-0.16	0.82	0.85 (0.17 - 4.27)	0.4%	0.6%	0.85	0.96	-0.16	0.82	0.85 (0.17 - 4.27)
SETX	ALS	40	4.0%	3.6%	0.35	0.70	0.27	0.29	1.31 (0.75 - 2.29)	4.9%	6.2%	0.62	0.96	-0.12	0.24	0.89 (0.55 - 1.43)
SQSTM1	ALS	5	0.7%	0.8%	0.78	0.92	-0.18	0.63	0.84 (0.24 - 2.88)	0.7%	0.8%	0.78	0.96	-0.18	0.63	0.84 (0.24 - 2.88)
TBK1	ALS	5	0.7%	0.0%	0.004	0.054	2.56	0.90	12.9 (2.22 - 75.07)	0.7%	0.0%	0.004	0.054	2.56	0.90	12.91 (2.22 - 75.08)
UNC13A	ALS	11	1.4%	1.6%	0.38	0.70	0.41	0.46	1.5 (0.61 - 3.72)	1.4%	1.6%	0.38	0.88	0.41	0.46	1.5 (0.61 - 3.72)
GRN	DEM	19	1.8%	2.1%	0.50	0.50	-0.27	0.40	0.76 (0.35 - 1.68)	1.8%	2.1%	0.50	0.50	-0.27	0.40	0.76 (0.35 - 1.68)
MAPT	DEM	23	3.6%	3.1%	0.33	0.50	0.31	0.32	1.36 (0.73 - 2.54)	5.4%	4.5%	0.26	0.50	0.30	0.27	1.35 (0.8 - 2.28)
PRKRA	DYS	15	2.2%	1.3%	0.20	0.20	0.53	0.42	1.7 (0.75 - 3.87)	10.7%	9.2%	0.38	0.38	0.17	0.19	1.18 (0.81 - 1.73)
TBP	HD	27	2.9%	3.3%	0.50	0.50	-0.22	0.32	0.8 (0.43 - 1.52)	9.4%	8.2%	0.42	0.42	0.15	0.18	1.16 (0.81 - 1.65)
ATM	OTHERS	29	4.0%	4.5%	0.80	0.86	-0.07	0.27	0.94 (0.55 - 1.58)	5.6%	5.6%	0.68	0.86	0.10	0.24	1.1 (0.69 - 1.76)
BTNL2	OTHERS	17	0.0%	0.1%	0.55	0.86	-1.36	2.27	0.26 (0 - 21.69)	6.2%	6.4%	0.66	0.86	-0.10	0.23	0.9 (0.58 - 1.41)
CLCN6	OTHERS	11	1.0%	1.0%	0.79	0.86	0.16	0.59	1.17 (0.37 - 3.69)	1.0%	1.0%	0.79	0.86	0.16	0.59	1.17 (0.37 - 3.69)
CSF1R	OTHERS	21	2.5%	2.3%	0.23	0.81	0.42	0.35	1.53 (0.76 - 3.05)	3.8%	3.6%	0.12	0.86	0.45	0.29	1.57 (0.89 - 2.77)
CTSC	OTHERS	7	0.6%	0.8%	0.53	0.86	-0.38	0.61	0.68 (0.21 - 2.25)	0.6%	0.8%	0.53	0.86	-0.38	0.61	0.68 (0.21 - 2.25)
DNMT1	OTHERS	15	1.2%	1.0%	0.86	0.86	0.10	0.56	1.1 (0.37 - 3.28)	5.2%	5.0%	0.42	0.86	0.21	0.26	1.23 (0.74 - 2.06)
EP300	OTHERS	17	2.2%	2.9%	0.30	0.81	-0.38	0.36	0.69 (0.34 - 1.39)	2.2%	2.9%	0.30	0.86	-0.38	0.36	0.69 (0.34 - 1.39)
NOTCH3	OTHERS	25	3.8%	4.3%	0.85	0.86	0.05	0.28	1.06 (0.61 - 1.83)	5.9%	6.0%	0.95	0.95	0.02	0.24	1.02 (0.64 - 1.62)
PANK2	OTHERS	12	1.2%	0.7%	0.09	0.80	0.95	0.56	2.59 (0.86 - 7.83)	1.8%	2.4%	0.55	0.86	-0.23	0.39	0.79 (0.37 - 1.7)
PLA2G6	OTHERS	13	1.2%	1.4%	0.41	0.86	-0.41	0.50	0.67 (0.25 - 1.76)	1.2%	1.4%	0.41	0.86	-0.41	0.50	0.67 (0.25 - 1.76)
PRNP	OTHERS	7	0.6%	0.2%	0.31	0.81	0.83	0.82	2.29 (0.46 - 11.38)	2.3%	1.8%	0.15	0.86	0.56	0.39	1.75 (0.82 - 3.74)
PRRT2	OTHERS	5	0.1%	0.5%	0.12	0.80	-1.54	1.00	0.21 (0.03 - 1.52)	1.3%	1.3%	0.79	0.86	-0.13	0.47	0.88 (0.35 - 2.21)
SPAST	OTHERS	8	0.4%	0.6%	0.68	0.86	-0.32	0.78	0.72 (0.16 - 3.32)	0.4%	0.6%	0.68	0.86	-0.32	0.78	0.72 (0.16 - 3.32)

MAF, minor allele frequency; T1R, Type-1 reaction; FDR, Benjamini–Hochberg False Discovery Rate; SE, Standard error; AD, Alzheimer's disease; ALS, amyotrophic lateral

Key Resource Table.

RESOURCE TYPE	RESOURCE NAME	SOURCE	IDENTIFIER	NEW/ REUSE
Dataset	NGS data of leprosy cases	Restricted	Not shareable due to consent restrictions	reuse
Chemical, peptide, or recombinant protein	TruSeq Neurodegeneration Panel	Illumina	cat# 20021356	new
Chemical, peptide, or recombinant protein	HiSeq 4000	Illumina	cat# PE-410-1001	new
Software/code	Bash codes	Github	https://github.com/vinimfava/Neurodegenerative_panel	new
